# Developmental stages in microbiota, bile acids, and clostridial species in healthy puppies

**DOI:** 10.1111/jvim.15928

**Published:** 2020-10-13

**Authors:** Amanda B. Blake, Annalis Cigarroa, Hannah L. Klein, Mohammad R. Khattab, Theresa Keating, Patti Van De Coevering, Jonathan A. Lidbury, Jörg M. Steiner, Jan S. Suchodolski

**Affiliations:** ^1^ Gastrointestinal Laboratory ‐ Texas A&M University College Station Texas USA; ^2^ Guide Dogs for the Blind San Rafael California USA

**Keywords:** canine, *Clostridium difficile*, *Clostridium hiranonis*, microbiota

## Abstract

**Background:**

The fecal microbiota, fecal bile acid concentrations, and abundance of *Clostridium perfringens* and *Clostridium difficile* are altered in acute and chronic gastrointestinal disease in adult dogs. However, less is known in young puppies.

**Hypothesis/Objectives:**

To determine composition of the fecal microbiota, assess development of fecal bile acid profiles, and determine the abundance of Clostridial species in puppies, young adult dogs, and adult dogs.

**Animals:**

Healthy puppies from a whelping kennel (n = 53) and healthy client‐owned dogs <1 year old (n = 20) were separated into 6 age groups, then compared to client‐owned dogs over 1 year of age (n = 13).

**Methods:**

Prospective observational study. Naturally voided fecal samples were analyzed by quantitative polymerase chain reaction to measure bacterial abundances. Fecal bile acids were quantified using gas chromatography‐mass spectrometry.

**Results:**

Puppies up to 5 to 6 weeks of age had increased Dysbiosis Index (median [min‐max]: 5.39 [1.32‐8.6], *P* < .001), increased abundance of *C. difficile* (4.1 [0.01‐4.85] log DNA, *P* < .001), decreased secondary bile acid concentrations (0.61 [0.28‐5.06] μg/mg, *P* = .006), and decreased abundance of *C. hiranonis* (0.84 [0.01‐6.71], *P* = .005) compared to adult dogs (−4.62 [−8.36 to −0.61], 0.01 [0.01‐0.01], 4.12 [0.32‐8.94], and 6.02 [5.06‐7.00], respectively). Secondary bile acid concentration positively correlated with *C*. *hiranonis* abundance (ρ = 0.77; *P* < .001).

**Conclusions and Clinical Importance:**

The increase in secondary bile acids and simultaneous decrease of *C. difficile* and *C. perfringens* after 5 to 6 weeks of age warrants further investigation into regulatory impacts that secondary bile acids could have on clostridial species in dogs.

**Abbreviations**
CA: cholic acid
CDCA: chenodeoxycholic acid
CPE: Clostridium perfringens enterotoxin gene
DCA: deoxycholic acid
DI: dysbiosis index
GI: gastrointestinal
LCA: lithocholic acid
qPCR: quantitative polymerase chain reaction
UDCA: ursodeoxycholic acid

## 1 INTRODUCTION

The gastrointestinal (GI) microbiome is critical to the health of young dogs as it plays several roles, including the maturation of the immune system, protection against enteropathogens, and utilization of nutrients.^1^, ^2^ While there are numerous studies pertaining to the microbiome in adult dogs, few describe the postnatal development of the GI microbiome.^3^, ^4^, ^5^ The microbiome increases in species richness beginning on day 2 after birth, and a major shift occurs from predominantly Firmicutes on day 2 to a codominance of Bacteroidetes, Fusobacteria, and Firmicutes by day 21 of age in dogs.^3^ Further characterization is merited, as development of the microbiome is important for the health of the developing puppy.

Alteration of the fecal microbiota is common in adult dogs with GI diseases.^6^, ^7^, ^8^, ^9^, ^10^, ^11^ The fecal dysbiosis index (DI) is a tool to evaluate the GI microbiota during disease and monitor response to treatment.^12^ The DI is a rapid quantitative polymerase chain reaction (qPCR)‐based assay that measures total bacteria and 7 key bacterial taxa (*Fusobacterium*, *Clostridium hiranonis*, *Faecalibacterium*, *Streptococcus*, *Turicibacter*, *Blautia*, and *Escherichia coli*) which were selected for having the highest discriminatory power in identifying dysbiosis in adult dogs with chronic enteropathies. A DI below zero is indicative of a healthy microbiota in dogs. Importantly, no studies have examined if the same index is applicable in young dogs. Based on previous studies,^3^, ^5^ puppies have an increased abundance of *E. coli* and a decreased abundance of *Faecalibacterium*, which would be expected to lead to an apparently increased DI in young healthy dogs.

Additional bacterial groups used to evaluate GI diseases in adult dogs include *Clostridium difficile*, *Clostridium perfringens*, *Salmonella* spp., and *Campylobacter jejuni*. Although multiple studies show these bacterial groups and their respective toxins to be present in healthy adult dogs and dogs with GI disease, little is known about their presence in young healthy dogs.^13^, ^14^, ^15^, ^16^, ^17^, ^18^, ^19^

Bile acids are of growing interest in study of GI disease in dogs. Bile acids aid in digestion and absorption of lipids in the GI tract, but also serve as signaling molecules. Primary bile acids are converted to secondary bile acids by bacteria with 7α‐dehydroxylation capabilities. Secondary bile acids inhibit growth and germination of *C. difficile* in humans,^20^, ^21^ and it is expected that the same relationship exists in dogs. Secondary bile acids also inhibited growth of isolates from dogs of *E. coli* and *C. perfringens* in vitro.^22^ Conversely, *Clostridium hiranonis* has 7α‐dehydroxylation activity,^23^ is part of the DI of dogs,^12^ and is likely the main contributor to the secondary bile acid pool in dogs.^6^, ^24^ Therefore, the association between the development of bile acid profiles and the development of the microbiota, and the potential influence of bile acids on *Clostridial* species in growing puppies needs further examination.

The objective of our study was to determine at which age the fecal DI resembles that of adult dogs and to assess the development of fecal bile acid profiles and their correlation with abundances of *C*. *hiranonis* and *C. difficile* in puppies of different ages.

## 2 MATERIALS AND METHODS

### 2.1 Animal enrollment

Puppies equal to or less than 10 weeks of age were enrolled from the Guide Dogs for the Blind, Inc in San Rafael, California (n = 53), and freely passed fecal samples (total n = 58) were collected. For the purpose of our study, fecal samples were separated into 5 groups based on the age of the puppy rounded up to the nearest week (Table 1): 1 to 2 weeks (n = 14), 3 to 4 weeks (n = 14), 5 to 6 weeks (n = 13), 7 to 9 weeks (n = 16), and 10 to 16 weeks (n = 1). Five of the puppies were represented in 2 age groups. All of these puppies were Golden Retrievers, Labrador Retrievers, or Golden Labrador mixes. They were born in a whelping kennel, and all puppies were fed the same diet plan during their entire time in the kennels. Starting at 3 weeks of age (21 days), puppies were offered Purina Pro Plan Large Breed Puppy Chicken & Rice formula in a 1 : 1.2 kibble to water ratio 3 times daily. At 4 weeks and 4 days to 4 weeks and 5 days (32‐33 days) of age, puppies were transitioned to a 3 : 1 kibble to water ratio and intermittently separated from brood to encourage weaning. By 6 weeks of age, puppies were fully weaned and then moved to a puppy kennel. Biosecurity measures were present in each kennel to prevent outside pathogens from entering. At 8 to 10 weeks of age, the puppies were sent to homes across the Western United States to be raised and socialized by volunteers until returning for formal guide dog training. Feces were stored at −20°C at the whelping facility until the end of collection, upon which they were shipped on dry ice to the Gastrointestinal Laboratory at Texas A&M University. Upon arrival to the laboratory, samples were inventoried while remaining on dry ice over the course of 3 hours and then transferred to −80°C storage until further processing.

**TABLE 1:** Animal enrollment and data (if available) for dogs included in the study

Age group	Sample number	Source	Sex (female/male)
1‐2 weeks	14	Guide dogs	6/8
3‐4 weeks	14	Guide dogs	8/5
5‐6 weeks	13	Guide dogs	8/5
7‐9 weeks	17	Guide dogs (16) and Client‐owned dogs (1)	9/8
10‐16 weeks	11	Guide dogs (1) and client‐owned dogs (10)	7/4
20‐48 weeks	11	Client‐owned dogs	6/5
>52 weeks	13	Client‐owned dogs	7/6

*Note*: Seven dogs were represented in 2 age groups.

Since further sampling was not able to be completed on the puppies once they left the whelping facility, 33 additional healthy dogs aged 8 weeks and older were enrolled in College Station, Texas. Freely passed fecal samples (total n = 35) were collected and separated into 4 age groups based on the age of the dog at the time of sampling: 7 to 9 weeks (n = 1), 10 to 16 weeks (n = 10), 20 to 48 weeks (n = 11), and >52 weeks (n = 13). Two dogs were represented in 2 age groups. These dogs were client‐owned, lived indoors, and were fed a variety of diets. Recruitment was conducted at puppy training classes, dog parks, and through social media at the veterinary school. Owners verbally consented to the use of their pet's feces in research and filled out a questionnaire to provide information about their dog (Supporting Information: Questionnaire 1). Inclusion criteria for all dogs included the absence of clinical signs and no antibiotic usage in the previous 6 months. Feces utilized in the study were stored at −80°C until processing.

Institutional animal care and use committee approval was not necessary for our study because all fecal samples used were naturally voided.

### 2.2 DNA isolation and qPCR

DNA was extracted from approximately 100 mg feces using a commercially available kit (PowerSoil DNA Isolation Kit, MOBIO Laboratories, Inc, Carlsbad, California) following manufacturer's instructions. Then, the DNA was normalized for concentration on a 96‐well plate utilizing DNA concentration and purity obtained on a Nanodrop 1000 (Thermo Scientific, Rockford, Illinois).

The abundances of selected bacterial groups were assessed by qPCR assays using published oligonucleotides described in Table 2. All samples were analyzed in duplicate on a commercially available qPCR thermal cycler (CFX96TM, Bio‐Rad Laboratories, California), and data expressed as LogSQ, or log target DNA (fg) per 10 ng starting quantity of total DNA.

**TABLE 2:** Primers and cycling conditions used in qPCR assays

Target	Primer sequences (5′ ‐ 3′)	Initial denaturing, # cycles	Denaturing	Annealing	Extension	Reference
Universal	F‐CCTACGGGAGGCAGCAGT R‐ATTACCGCGGCTGCTGG	98°C, 2 min, 35	98°C, 5 s	59°C, 5 s		25
*Faecalibacterium* spp.	F‐GAAGGCGGCCTACTGGGCAC R‐GTGCAGGCGAGTTGCAGCCT	98°C, 2 min, 40	98°C, 5 s	60°C, 5 s		26
*Turicibacter* spp.	F‐CAGACGGGGACAACGATTGGA R‐TACGCATCGTCGCCTTGGTA	98°C, 2 min, 40	98°C, 3 s	57°C, 3 s		27
*Streptococcus* spp.	F‐TTATTTGAAAGGGGCAATTGCT R‐GTGAACTTTCCACTCTCACAC	95°C, 2 min, 40	95°C, 5 s	54°C, 10 s		28
*Escherichia coli*	F‐GTTAATACCTTTGCTCATTGA R‐ACCAGGGTATCTAATCCTGTT	98°C, 2 min, 40	98°C, 3 s	55°C, 3 s		29
*Blautia* spp.	F‐TCTGATGTGAAAGGCTGGGGCTTA R‐GGCTTAGCCACCCGACACCTA	98°C, 2 min, 40	98°C, 4 s	56°C, 4 s		27
*Fusobacterium* spp.	F‐KGGGCTCAACMCMGTATTGCGT R‐TCGCGTTAGCTTGGGCGCTG	98°C, 2 min, 40	98°C, 4 s	50.5°C, 4 s		27
*Clostridium hiranonis*	F‐AGTAAGCTCCTGATACTGTCT R‐AGGGAAAGAGGAGATTAGTCC	95°C, 3 min, 40	95°C, 30 s	59°C, 5 s		30
*C perfringens* NetF toxin	F‐AACAATATGTACAGGTATAACT R‐TTGATAGGTATAATATGGTTCT	98°C, 2 min, 40	98°C, 30 s	55°C, 30 s		16
*C perfringens* 16S rRNA	F‐CGCATAACGTTGAAAGATGG R‐CCTTGGTAGGCCGTTACCC P‐TCATCATTCAACCAAAGGAGCAATCC	94°C, 10 min, 45	94°C, 10 s	58°C, 20 s	70°C, 10 s	31
*C difficile* 16S rRNA	F‐TTGAGCGATTTACTTCGGTAAAGA R‐TGTACTGGCTCACCTTTGATATTCA P‐CCACGCGTTACTCACCCGTCCG	95°C, 2 min, 40	95°C, 5 s	61°C, 10 s		32
*C perfringens* enterotoxin	F‐AACTATAGGAGAACAAAATACAATAG R‐TGCATAAACCTTATAATATACATATTC P‐TCTGTATCTACAACTGCTGGTCCA	95°C, 2 min, 40	95°C, 5 s	55°C, 10 s		33
*Salmonella* spp.	F‐GCAATTACAGGAACAGACGCT R‐CCTGACGCCCGTAAGAGA P‐TAAAACTTCGCCATACCAGCCAGACA	95, 2 min, 40	95, 5 s	60, 10 s		34
*Campylobacter jejuni*	F‐TTAATGACGCGGTAAAAGTAACTATGG R‐TGCTTGGAGCACCAAAGCT P‐CCAAGAGGACGCAATGT	95, 2 min, 40	95, 5 s	52.5, 20 s		35

Abbreviations: F, forward primer; P, probe; qPCR, quantitative polymerase chain reaction; R, reverse primer.

The qPCR assays for total bacteria, *Faecalibacterium* spp., *Turicibacter* spp., *Streptococcus* spp., *Escherichia coli*, *Blautia* spp., *Fusobacterium* spp., *Clostridium hiranonis*, and *C. perfringens* NetF toxin gene were performed using previously published cycling and annealing conditions described in Table 2.^16^, ^25^, ^26^, ^27^, ^28^, ^29^, ^30^ Briefly, SYBR Green based reaction mixtures (total 10 μL) contained 5 μL of SsoFast EvaGreen Supermix (Bio‐Rad Laboratories), 2.2 μL of PCR water, 0.4 μL of each primer (final concentration: 400 nM), and 2 μL of normalized DNA (final concentration: 5 ng/μL). A melt curve analysis, with increments of 0.5°C from 65°C to 95°C for 5 seconds each, was performed after the amplification cycles.

The qPCR assay for the *C. perfringens* 16S rRNA gene was performed as previously described.^13^, ^31^ Briefly, TaqMan based reaction mixtures (total 10 μL) contained 5 μL of SsoAdvanced Universal Probes Supermix, 2.2 μL of PCR water, 0.3 μL of each primer (final concentration: 300 nM), 0.2 μL of the probe (final concentration: 200 nM), and 2 μL of normalized DNA (final concentration: 5 ng/μL). The qPCR cycling and annealing conditions are described in Table 2.

The qPCR assays for the *Clostridium difficile* 16S rRNA gene, *Clostridium perfringens* enterotoxin gene (CPE), *Salmonella* spp., and *Campylobacter jejuni* were performed as previously described.^13^, ^32^, ^33^, ^34^, ^35^ Briefly, TaqMan based reaction mixtures (total 10 μL) contained 5 μL of SsoAdvanced Universal Probes Supermix (Bio‐Rad Laboratories), 2.35 μL of PCR water, 0.25 μL of each forward and reverse primer (final concentration: 250 nM), 0.15 μL of the probe (final concentration: 150 nM), and 2 μL of normalized DNA (final concentration: 5 ng/μL). The qPCR cycling and annealing conditions are described in Table 2.

### 2.3 Dysbiosis index

The fecal DI was calculated from the abundance of key bacterial taxa as previously described.^12^ Briefly, the abundances of total bacteria, *Faecalibacterium* spp., *Turicibacter* spp., *Streptococcus* spp., *Escherichia coli*, *Blautia* spp., *Fusobacterium* spp., and *Clostridium hiranonis* are combined using a mathematical algorithm and reported as the DI. A DI below zero is indicative of a normal adult microbiota in dogs.

### 2.4 *Clostridium* spp. toxins

Fecal samples that tested positive for *C. difficile* 16S rRNA through qPCR were subjected to further screening with the C. Diff Quik Chek Complete test (TechLab, Inc, Blackburg, Virginia) for the detection of *C. difficile* glutamate dehydrogenase antigen and toxins A and B when leftover feces were available.

Fecal samples that tested positive for the *C. perfringens* enterotoxin gene through qPCR were subjected to further screening with an ELISA based *C. perfringens* Enterotoxin Test (TechLab Inc) for the detection of enterotoxin produced by *C. perfringens* when leftover feces were available.

### 2.5 Quantification of fecal bile acids

The following unconjugated bile acids were quantified by an in‐house targeted gas chromatography‐mass spectrometry assay utilizing previously described methods^6^: cholic acid (CA), chenodeoxycholic acid (CDCA), deoxycholic acid (DCA), lithocholic acid (LCA), and ursodeoxycholic acid (UDCA).

### 2.6 Statistical analysis

Statistical analysis was performed using statistical software packages (JMP Pro version 14, SAS Institute, Inc, Cary, North Carolina; and Prism version 8, GraphPad Software, Inc, La Jolla, California). Some data showed a nonparametric distribution based on the Shapiro‐Wilk normality test results. Therefore, nonparametric testing was used for further analysis. Kruskal‐Wallis tests were used to assess differences in measured variables across the age groups. Dunn's multiple comparisons post‐tests were used to identify differences between each of the 6 age groups <1 year of age and healthy adult dogs >1 year of age. Spearman's correlation analysis was performed between *Clostridial* species abundances and total secondary bile acid concentrations. Significance was set at *P* < .05.

## 3 RESULTS

Statistical test results for all variables are listed in Table S1.

### 3.1 Dysbiosis index

**FIGURE 1:**
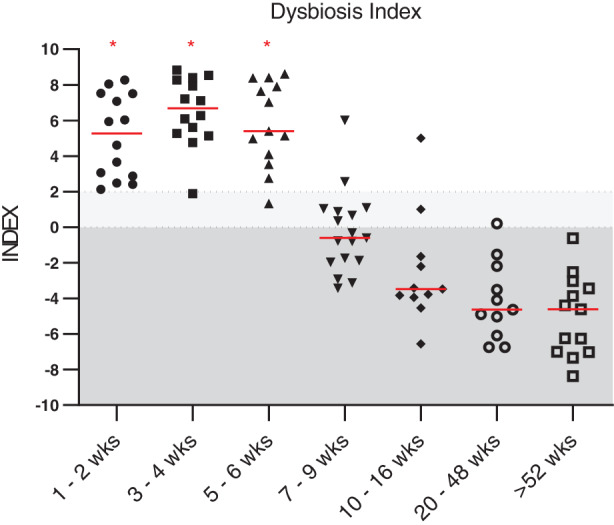
Fecal dysbiosis index of dogs age 1 to 2 weeks (n = 14), 3 to 4 weeks (n = 14), 5 to 6 weeks (n = 13), 7 to 9 weeks (n = 17), 10 to 16 weeks (n = 11), 20 to 48 weeks (n = 11), and adult dogs (ie, dogs >52 weeks of age; n = 13). Dysbiosis index was significantly increased in dogs age 1 to 2 weeks, 3 to 4 weeks, and 5 to 6 weeks (*P* < .001 for all) when compared to adult dogs. Dysbiosis index was not significantly different from adult dogs at 7 to 9 weeks of age (*P* = .05), 10 to 16 weeks of age (*P* > .99), and 20 to 48 weeks of age (*P* > .99). Red lines indicate medians and red asterisks indicate significance in comparison to adult dogs (*P* < .05). Dark shaded area (values <0) represents reference interval, and light shaded area (0‐2) represents an equivocal range. wks, weeks

**FIGURE 2:**
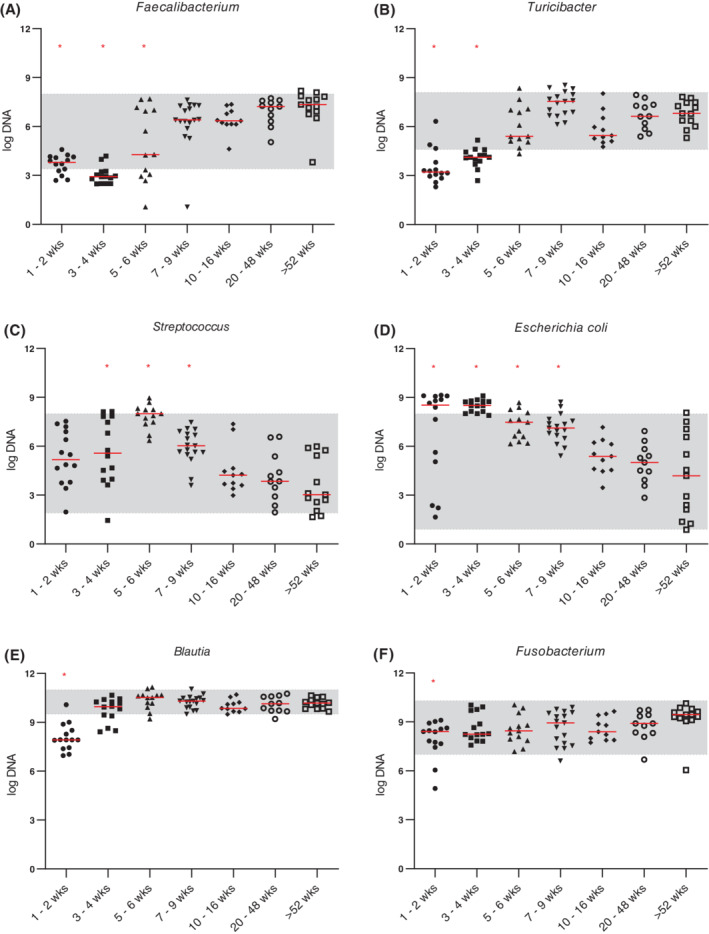
Abundances (log DNA) of bacterial groups included in the dysbiosis index for dogs age 1 to 2 weeks (n = 14), 3 to 4 weeks (n = 14), 5 to 6 weeks (n = 13), 7 to 9 weeks (n = 17), 10 to 16 weeks (n = 11), 20 to 48 weeks (n = 11), and adult dogs (ie, dogs >52 weeks of age; n = 13). A, Abundance of *Faecalibacterium* was significantly decreased in dogs age 1 to 2 weeks (*P* < .001), 3 to 4 weeks (*P* < .001), and 5 to 6 weeks (*P* = .02) when compared to adult dogs. B, Abundance of *Turicibacter* was significantly decreased in dogs age 1 to 2 weeks and 3 to 4 weeks (both *P* < .001) compared to adult dogs. C, Abundance of *Streptococcus* was significantly increased in dogs age 3 to 4 weeks (*P* = .03), 5 to 6 weeks (*P* < .001), and 7 to 9 weeks (*P* = .007) compared to adult dogs. D, Abundance of *Escherichia coli* was significantly increased in dogs age 1 to 2 weeks (*P* = .005), 3 to 4 weeks (*P* < .001), 5 to 6 weeks (*P* = .02), and 7 to 9 weeks (*P* = .03) compared to adult dogs. E, Abundance of *Blautia* and, F, *Fusobacterium* was significantly decreased in dogs 1 to 2 weeks of age (*P* < .001 and *P* = .003, respectively) compared to adult dogs. Red lines indicate medians and red asterisks indicate significance in comparison to adult dogs (*P* < .05). Shaded areas correspond to reference intervals. wks, weeks

**FIGURE 3:**
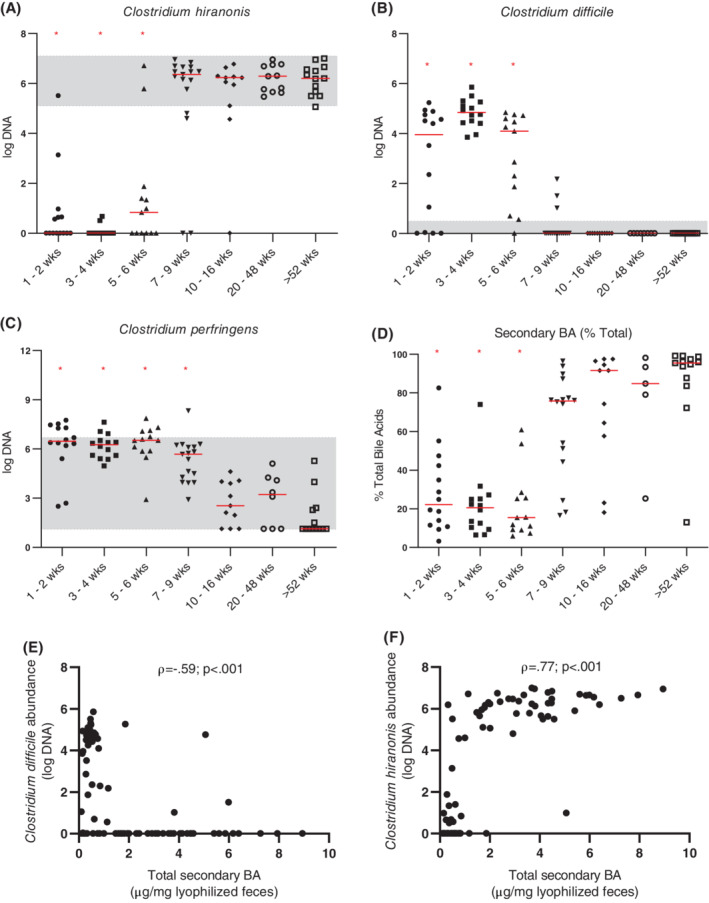
Abundances (log DNA) of *Clostridial* spp. (A, B, C), percentage of secondary bile acids as a percent of total measured bile acids (D), and Spearman's correlations of total secondary bile acids, *Clostridium difficile* (E), and *Clostridium hiranonis* (F). Graphs (A‐D) depict dogs age 1 to 2 weeks (n = 14 for all), 3 to 4 weeks (n = 14 for all), 5 to 6 weeks (n = 13 for all), 7 to 9 weeks (n = 17 for A‐C, n = 16 for D), 10 to 16 weeks (n = 11 for all), 20 to 48 weeks (n = 11 for A, n = 8 for B and C, n = 5 for D), and adult dogs (ie, dogs >52 weeks of age; n = 13 for all). Abundance of *Clostridium hiranonis* (A) was significantly decreased in dogs age 1 to 2 weeks (*P* < .001), 3 to 4 weeks (*P* < .001), and 5 to 6 weeks (*P* = .005) when compared to adult dogs. Abundance of *Clostridium difficile* (B) was significantly increased in dogs age 1 to 2 weeks, 3 to 4 weeks, and 5 to 6 weeks (*P* < .001 for all) compared to adult dogs. Abundance of *Clostridium perfringens* (C) was significantly increased in dogs age 1 to 2 weeks (*P* < .001), 3 to 4 weeks (*P* < .001), 5 to 6 weeks (*P* < .001), and 7 to 9 weeks (*P* = .002) compared to adult dogs. Percentage of secondary bile acids was significantly decreased in dogs age 1 to 2 weeks, 3 to 4 weeks, and 5 to 6 weeks (*P* < .001 for all) compared to adult dogs. Total secondary bile acid concentration was negatively correlated with *C. difficile* abundance (E; ρ = −0.59) and positively correlated with *C. hiranonis* abundance (F; ρ = 0.77; *P* < .001 and n = 86 for both). Red lines indicate medians and red asterisks indicate significance in comparison to adult dogs (*P* < .05). Shaded areas correspond to reference intervals. ρ, Spearman's rho; BA, bile acid; wks, weeks

The DI was significantly higher in puppies that were 1 to 2 weeks, 3 to 4 weeks, and 5 to 6 weeks of age (*P* < .001) when compared to adult dogs (dogs >52 weeks old; Figure 1). Dogs that were 1 to 2 weeks, 3 to 4 weeks, and 5 to 6 weeks old also showed a decreased abundance of *Faecalibacterium* when compared to adult dogs (*P* < .001, *P* < .001, and *P* = .02, respectively; Figure 2). Abundance of *Turicibacter* was decreased in puppies 1 to 2 and 3 to 4 weeks of age (*P* < .001) compared to adult dogs (Figure 2). Abundance of *Streptococcus* was increased in puppies that were 3 to 4, 5 to 6, and 7 to 9 weeks of age (*P* = .03, *P* < .001, and *P* = .007, respectively), and the abundance of *E. coli* was significantly increased in puppies that were 1 to 2, 3 to 4, 5 to 6, and 7 to 9 weeks old (*P* = .005, *P* < .001, *P* = .02, and *P* = .03, respectively) compared to adult dogs (Figure 2). Abundances of *Blautia* and *Fusobacterium* were significantly decreased in puppies that were 1 to 2 weeks of age (*P* < .001 and *P* = .003, respectively) when compared to adult dogs (Figure 2). Abundance of *C. hiranonis* was significantly decreased in puppies that were 1 to 2, 3 to 4, and 5 to 6 weeks old (*P* < .001, *P* < .001, and *P* = .005, respectively) when compared to adult dogs (Figure 3). Abundance of total bacteria was increased in puppies that were 3 to 4 weeks and 5 to 6 weeks old (*P* = .05 and *P* = .02, respectively) when compared to adult dogs (Figure S1).

### 3.2 *Clostridium* spp., *Salmonella*, and *Campylobacter jejuni*

None of the fecal samples from any of the dogs evaluated contained the NetF toxin gene. The abundance of *C. perfringens* was significantly increased in puppies that were 1 to 2, 3 to 4, 5 to 6, and 7 to 9 weeks old (*P* < .001, *P* < .001, *P* < .001, and *P* = .002, respectively) when compared to the healthy adult dogs (Figure 3). The abundance of *C. difficile* was significantly increased in puppies that were 1 to 2, 3 to 4, and 5 to 6 weeks of age (*P* < .001) when compared to adult dogs (Figure 3). Out of 87 samples total, 40 tested positive for *C. difficile* by qPCR. Of those 40 samples, the C. Diff Quik Chek Complete test was performed on 30 samples for which leftover sample material was available. All 30 samples were toxin negative, and 27 out of 30 samples were antigen positive. Additionally, 8 out of 87 samples were positive for CPE by qPCR. Of those 8 samples, 6 samples were tested for *C. perfringens* enterotoxin by ELISA, and none of the 6 samples tested positive. The abundance of CPE was decreased in puppies that were 1 to 2 or 5 to 6 weeks of age (*P* = .01 and *P* = .02, respectively) when compared to the adult dogs (Figure S2). *Clostridium difficile* abundance was negatively correlated with the abundance of *C. hiranonis* (ρ = −0.7; *P* < .001; Figure 3).

*Salmonella* and *C. jejuni* assays were only able to be performed on adult dog samples and samples from dogs 9 weeks of age and younger due to limited amount of feces (n = 70 total). Two samples from puppies that were 5 to 6 weeks of age and 2 samples from puppies that were 7 to 9 weeks of age were positive for *Salmonella*, but all samples were negative for *C. jejuni*.

### 3.3 Fecal unconjugated bile acids

**FIGURE 4:**
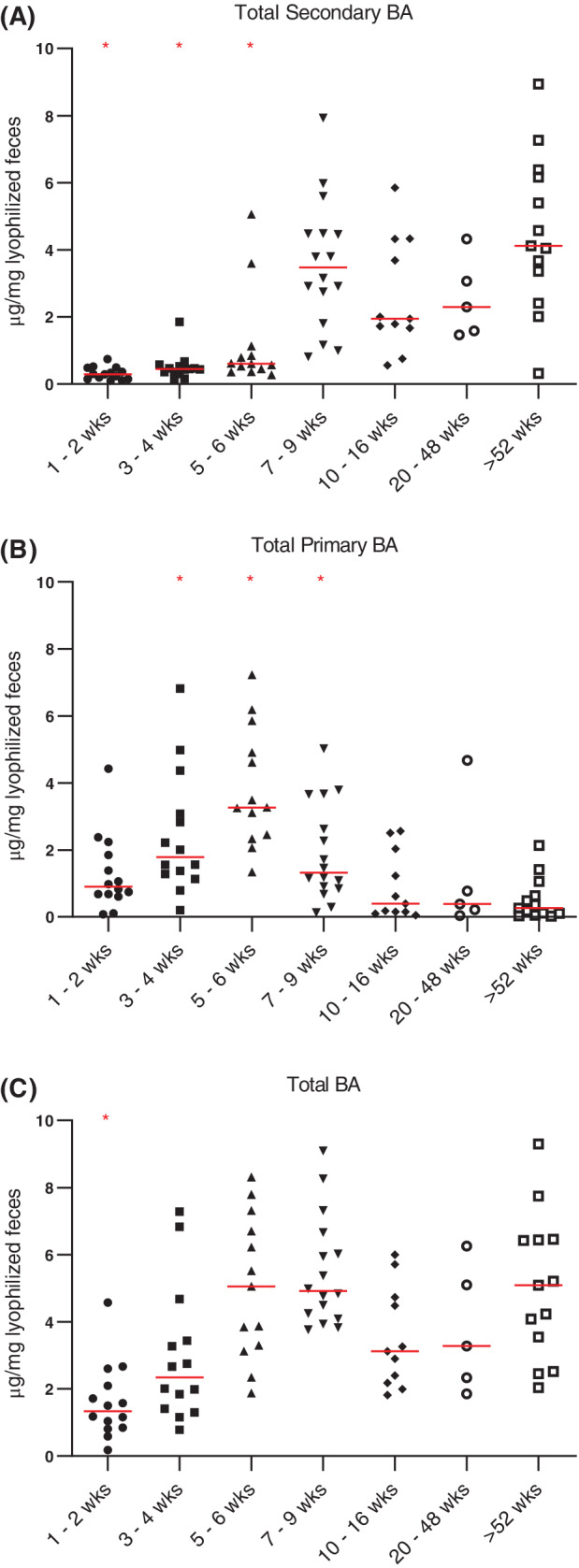
Concentration of fecal bile acids (μg/mg lyophilized feces) for dogs age 1 to 2 weeks (n = 14), 3 to 4 weeks (n = 14), 5 to 6 weeks (n = 13), 7 to 9 weeks (n = 16), 10 to 16 weeks (n = 11), 20 to 48 weeks (n = 5), and adult dogs (ie, dogs >52 weeks of age; n = 13). Total secondary BA represents the sum of concentrations of deoxycholic acid, lithocholic acid, and ursodeoxycholic acid. Total primary BA represents the sum of cholic acid and chenodeoxycholic acid concentrations. Total BA represents the sum of total primary and total secondary bile acid concentrations. Concentration of secondary bile acids (A) was significantly decreased in dogs age 1 to 2 weeks (*P* < .001), 3 to 4 weeks (*P* < .001), and 5 to 6 weeks (*P* = .006) when compared to adult dogs. Concentration of primary bile acids (B) was significantly increased in dogs age 3 to 4 weeks (*P* = .003), 5 to 6 weeks (*P* < .001), and 7 to 9 weeks (*P* = .02) compared to adult dogs. Concentration of total bile acids (C) was significantly decreased in dogs age 1 to 2 weeks compared to adult dogs (*P* < .001). Red lines indicate medians and red asterisks indicate significance in comparison to adult dogs (*P* < .05). BA, bile acid; wks, weeks

Due to limited amount of feces, fecal bile acid concentrations were not able to be obtained for 1 dog in the 7 to 9 week age group and 6 dogs in the 20 to 48 week age group. Graphs of concentrations of individual bile acids are provided in Figure S3. Fecal concentrations of CA were significantly increased in puppies that were 3 to 4 or 5 to 6 weeks of age (*P* = .003 and *P* < .001, respectively) when compared to the adult dogs. Fecal concentrations of CDCA were significantly increased in puppies that were 3 to 4, 5 to 6, and 7 to 9 weeks of age (*P* = .02, *P* < .001, and *P* < .001, respectively) when compared to the adult dogs. Fecal concentrations of LCA and DCA were significantly decreased in puppies that were 1 to 2 weeks (*P* < .001), 3 to 4 weeks (*P* < .001), or 5 to 6 weeks old (*P* = .05 and *P* = .001, respectively) when compared to the adult dogs. Fecal concentrations of UDCA were significantly increased in puppies that were 3 to 4, 5 to 6, or 7 to 9 weeks of age (*P* = .003, *P* < .001, and *P* < .001, respectively) when compared to the adult dogs. The total concentration of fecal primary bile acids (sum of CA and CDCA) was significantly increased in puppies that were 3 to 4, 5 to 6, or 7 to 9 weeks of age (*P* = .003, *P* < .001, and *P* = .02, respectively) when compared to adult dogs (Figure 4). Total concentration of fecal secondary bile acids (sum of DCA, LCA, and UDCA) was significantly decreased in puppies that were 1 to 2, 3 to 4, or 5 to 6 weeks of age (*P* < .001, *P* < .001, and *P* = .006, respectively) age groups when compared to adult dogs (Figure 4). The total concentration of fecal bile acids was decreased in puppies that were 1 to 2 weeks of age (*P* < .001) when compared to adult dogs (Figure 4). Total secondary bile acid concentration was negatively correlated with *C. difficile* abundance (ρ = −0.59; *P* < .001) and positively correlated with *C. hiranonis* abundance (ρ = 0.77; *P* < .001; Figure 3) across all tested samples.

## 4 DISCUSSION

Results of our study suggest that the fecal microbiome changes significantly with age in young dogs. The DI was significantly higher in puppies that were 1 to 2, 3 to 4, or 5 to 6 weeks of age (*P* < .001) when compared to the adult dogs. Several individual bacterial groups were also altered in puppies less than 7 to 9 weeks of age (Figures 1, 2, 3). These results are consistent with previous studies that show puppies under 6 weeks of age to have different fecal and GI microbial communities from adult dogs.^3^, ^4^, ^5^ The increased DI in young dogs appeared to be driven by an increased abundance of *E. coli* and a decreased abundance of anaerobic bacterial groups (ie, *Faecalibacterium*, *Turicibacter*, and *C. hiranonis*). As the abundance of *E. coli* decreased and that of *C. hiranonis*, *Faecalibacterium*, *Blautia*, and *Turicibacter* increased, the microbiome of puppies started to become similar to that of adult dogs and the DI was negative in older puppies. These results suggest that the DI might need a different cutoff value for differentiation of normobiosis and dysbiosis in puppies that are 6 weeks of age or younger.

In our study, we evaluated the fecal abundance of 4 bacterial species that are listed on commercially available fecal enteropathogen panels (ie, *C. difficile*, *C. perfringens*, *Salmonella*, and *Campylobacter jejuni*) in addition to evaluating fecal samples for *C. difficile* and *C. perfringens* toxins and toxin‐encoding genes.

*Clostridium perfringens* is an anaerobic, spore‐forming bacterium that is found in fecal samples of both healthy dogs and dogs with diarrhea.^13^, ^14^, ^36^ There have been no studies in puppies that investigate whether *C. perfringens* colonizes at an early age. Our results suggest that *C. perfringens* does colonize at an early age and that its abundance decreased over time. This could be because it is an opportunistic bacterium and its decrease could be associated with an increased competition for space. It is also possible that the increase in secondary bile acids over time is associated with a decrease in *C. perfringens* (see discussion below).^22^

*Clostridium perfringens* enterotoxin (CPE) is a virulence factor that is found in both healthy dogs and dogs with diarrhea.^13^, ^14^, ^36^ One study suggested that CPE plays no role in acute hemorrhagic diarrhea syndrome.^14^ While its presence in adult dogs has been established, it was unknown whether this toxin is expressed in puppies. Our results suggest that CPE is present with a 5% prevalence in dogs under 1 year of age. Further studies should be performed looking at this enterotoxin gene presence in young dogs with GI symptoms to determine if it may be a contributing factor in the pathogenesis of diarrhea.

NetF toxin is a novel toxin associated with *C. perfringens*. As it is commonly associated with acute hemorrhagic diarrhea syndrome in dogs^16^, we did not expect to find it in samples of healthy dogs. This expectation was confirmed by our data.

*Salmonella* and *Campylobacter jejuni* are well‐known zoonoses that have been isolated in both healthy dogs and dogs with diarrhea. The prevalence of *Salmonella* in our healthy cohort (0.6%) fell within the range previously reported for healthy dogs (0%‐3.6%)^37^, and there were no significant differences between puppies of any age and adult dogs. *Campylobacter jejuni* was not detected in any of the 70 fecal samples evaluated, regardless of age. Previous studies show a prevalence of *C. jejuni* of 0.8% to 14.7% in dogs, and suggest that it is more frequently isolated from dogs under 1 year of age.^35^, ^36^, ^38^, ^39^, ^40^, ^41^ However, this is the first study, to our knowledge, that has looked at dogs under 10 weeks of age for *C. jejuni*. Further studies with a larger number of dogs in this age group could give a more accurate estimation of population prevalence and association with disease.

*Clostridium difficile* is a human pathogen that causes GI disease. While dogs are carriers for this bacterium, their carriage is not clearly associated with GI disease.^15^ Additionally, while there is an association with increasing age and an increase in risk for fecal shedding^18^, puppies have yet to be included in such studies. Our results suggest that *C. difficile* is initially abundant and decreases after 5 to 6 weeks of age. Similarly, in human infants, colonization by *C. difficile* is more common than in adults, but rarely causes disease.^42^ In our cohort of dogs, 40 out of 87 fecal samples tested positive for *C. difficile* by qPCR. Of those 40 samples, we were able to measure antigen and toxin A/B in 30 samples. Three of the 30 samples were antigen negative. This discordance is likely because of the relatively low quantity of *C. difficile* 16S rRNA detected in those 3 samples. None of the 30 samples were positive for *C. difficile* toxin A or B, similar to what has been observed in adult healthy dogs.^14^ Toxigenic strains of *C. difficile* have been identified in adult dogs with GI diseases^17^; however, the prevalence of toxigenic strains in dogs under 6 months of age was unknown. Our study suggests that nontoxigenic *C. difficile* is common in healthy puppies under 6 weeks of age.

A decreased abundance of *C. difficile* in puppies 7 to 9 weeks of age and older closely mirrored an increased abundance of *C. hiranonis* (Figure 3). One potential explanation of this observation could be the relationship that both of these *Clostridial* species have with bile acids. *Clostridium hiranonis* plays a role in the conversion of primary to secondary bile acids^23^, and its presence was strongly correlated with concentration of secondary bile acids (Figure 3). Additionally, the growth and spore germination of *C. difficile* is inhibited by secondary bile acids,^20^, ^21^ and 1 recent study shows that this inhibition is dose‐dependent.^43^ Fecal concentrations of secondary bile acids in puppies up to 5 to 6 weeks of age in our study were significantly lower than those in adult dogs (Figure 4). The concurrent increase in the concentration of secondary bile acids in the feces and decrease of *C. difficile* abundance supports the hypothesis that such a relationship exists in dogs.

Furthermore, bile acids influence the growth of other bacterial groups as well. Secondary bile acids inhibit the growth of isolates from dogs of *E. coli* and *C. perfringens*, in vitro.^22^ Additionally, a deoxycholic acid supplemented diet reduces invasion of *C. perfringens* into the ileum in chickens with *C. perfringens‐*induced necrotic enteritis.^44^ The results of our study suggest that such an inhibitory relationship may exist in dogs, albeit with a slight delay, as *E. coli* and *C. perfringens* abundances remained significantly increased in puppies that were 7 to 9 weeks of age.

In addition to the relationship bile acids have with the microbiota, they can also have independent effects on the host. A disruption in the bile acid pool that leads to secretory diarrhea, also known as bile acid diarrhea, is implicated in 25% to 33% of human patients with chronic functional diarrhea.^45^ This could be a contributing factor to diarrhea in some dogs with GI disease, as previous studies show an increase in fecal primary bile acids.^6^, ^10^ Additionally, 18% of dogs with chronic diarrhea have higher than normal values of serum 7α‐hydroxy‐4‐cholesten‐3‐1, a marker of bile acid malabsorption.^46^ Dogs with chronic enteropathy also have decreased ileal expression of the apical sodium‐dependent bile acid transporter.^10^ Interestingly, we found that the fecal bile acid profiles of healthy puppies 5 to 6 weeks of age and younger resembled that of adult dogs with GI disease, with increased primary bile acid concentrations and decreased secondary bile acid concentrations. It remains unclear if this observation is due to the maturing microbial community, physical changes in puppies, or a change in the diet with weaning. Further studies should examine *Clostridial* species and fecal bile acids in puppies with diarrhea to see if there is an altered pattern of maturation.

Limitations of our study include a small number of samples from puppies that were 10 weeks to 1 year of age and a lack of follow‐up for the vast majority of samples. The investigators were not able to follow the dogs over time, but instead sampled dogs from different age groups. Seven dogs were sampled twice and represented in 2 age groups in the study. Dogs were enrolled at 2 different locations because the first location did not have dogs that were 10 weeks to 1 year of age. The variability in environments could also be considered a limitation, as dogs cohoused in kennels have a different environment from those in a single pet household. However, most observed changes occurred already within the first 5 to 6 weeks of life, when all dogs were still living in the same environment. Fecal samples were not tested for Giardia or Cryptosporidium beforehand, as these dogs did not display clinical symptoms of GI disease. Future studies should consider following a greater number of puppies, with and without diarrhea, throughout their development in a standardized setting.

## 5 CONCLUSION

Similar to the relationship seen in humans, it is likely that secondary bile acids play a role in regulating growth of *C. difficile* in dogs. Our study suggests a relationship between *C. difficile*, *C. hiranonis*, and fecal bile acid concentrations in dogs, and highlights the use of metabolites in conjunction with microbial communities to elucidate function in the GI tract.

## CONFLICT OF INTEREST DECLARATION

The following authors are either currently or within the last 3 years employed by the Gastrointestinal Laboratory at Texas A&M University that performs diagnostic testing on a fee‐for‐service basis: Amanda B. Blake, Annalis Cigarroa, Hannah L. Klein, Mohammad R. Khattab, Jonathan A. Lidbury, Joerg M. Steiner, and Jan S. Suchodolski.

## OFF‐LABEL ANTIMICROBIAL DECLARATION

Authors declare no off‐label use of antimicrobials.

## INSTITUTIONAL ANIMAL CARE AND USE COMMITTEE (IACUC) OR OTHER APPROVAL DECLARATION

Authors declare no IACUC or other approval was needed.

## HUMAN ETHICS APPROVAL DECLARATION

Authors declare human ethics approval was not needed for this study.

## Supporting information


**Questionnaire 1** A questionnaire collected at the time of sampling to gain health history, dietary information, and signalment of the patient. PDF format.

**Supplemental Table 1** A table listing statistical test results for all variables. Xlsx format.

**Supplemental Figure 1** Abundance (log DNA) of total bacteria. Red lines indicate medians and red asterisks indicate significance in comparison to adult dogs (ie, dogs >52 weeks of age; *P* < .05).

**Supplemental Figure 2** Abundance (log DNA) of *Clostridium perfringens* enterotoxin gene. Red lines indicate medians and red asterisks indicate significance in comparison to adult dogs (ie, dogs >52 weeks of age; *P* < .05).

**Supplemental Figure 3** Concentration of fecal bile acids (μg/mg lyophilized feces). Red lines indicate medians and red asterisks indicate significance in comparison to adult dogs (ie, dogs >52 weeks of age; *P* < .05).
