# Supplementary material for: Developmental stages in microbiota, bile acids, and clostridial species in healthy puppies
Source: J Vet Intern Med. 2020 Oct 13;34(6):2345–56. doi: 10.1111/jvim.15928 (PMC7694855; doi:10.1111/jvim.15928)
Supplement: Supplementary file 1 — Questionnaire 1 A questionnaire collected at the time of sampling to gain health history, dietary information, and signalment of the patient. PDF format. [file JVIM-34-2345-s001.pdf]

The fecal microbiome and metabolome in healthy dogs and cats

**Fecal sample**	Collection date:		Storage until delivered:	◯ room temp	◯ refrigerated		◯ frozen
**Fecal score <u>(lab use only)</u>**	1	2	3	4	5	6	7

Patient Information

**Today's date**		**Owner email:**
**Pet's name**		**Owner name:**

Signalment

**Species**	◯ dog ◯ cat	Breed:	
**Age (years)***			
**How long have you owned your pet?**		**Where is your pet primarily kept?**	
**Sex**	◯ female	◯ male	◯ spayed/neutered
**Body weight***	Kg	lbs	**Date of last vaccination:**
**BCS***	1 2 3 4 (ideal - dogs) 5 6 7 8 9		

* at sample collection

History of current condition

<u>At the time</u> the fecal sample was collected

**GI disease**	◯ no	◯ yes, acute<br>(GI signs, including weight loss, < 3 weeks)		◯ yes, chronic<br>(GI signs, including weight loss, > 3 weeks)		
**Appetite**	◯ normal	◯ decreased	◯ increased	◯ mild	◯ moderate	◯ severe
**Attitude/Activity**	◯ normal	◯ decreased		◯ mild	◯ moderate	◯ severe
**Vomiting**	◯ no	◯ less than 1x/week	◯ 1-2x/week	◯ more than 2x/week		
**Diarrhea**	◯ no	◯ less than 1x/week	◯ 1-2x/week	◯ more than 2x/week		
**Weight loss**	◯ none	◯ mild	◯ moderate	◯ severe		

**Any medications**	◯ none	◯ yes (please specify)
**Any antibiotic use in the last 30 days**	◯ none	◯ yes (please specify which antibiotic and for what condition)
**Any antibiotic use in the last 6 months**	◯ none	◯ yes (please specify which antibiotic and for what condition)
**Any antibiotic use ever**	◯ none	◯ yes (please specify which antibiotic and for what condition)
**Any pre- existing conditions/allergies**	◯ none	◯ yes (please specify)
**When did the first clinical sign(s) start**	about _______________ days/ weeks/month/years ago	

**Diet**	◯ Dry food (type: Purina Savor, Royal Canin Satiety, etc.)<br>____________________<br>◯ Wet food (type: Purina Savor, Royal Canin Satiety, etc.)<br>____________________<br>◯ Homemade diet (please include what you feed, ex: chicken, rice, etc.)<br>____________________<br>◯ Raw food (type: Countrypet Duck recipe, etc or what you feed, ex: chicken, carrots, etc.)<br>____________________<br>◯ Pre- or Pro- biotics (include brand name)<br>____________________<br>◯ Other, please explain:<br>____________________<br>◯**Length of time your pet has been on this diet/diets:**<br>____________________

Previous History

**Date (approximate)**						
**Appetite**	◯ normal	◯ decreased ◯ increased	◯ mild	◯ moderate	◯ severe	
**Attitude/Activity**	◯ normal	◯ decreased	◯ mild	◯ moderate	◯ severe	
**Vomiting**	◯ no	◯ less than 1x/week	◯ 1-2x/week	◯ more than 2x/week		
**Diarrhea**	◯ no	◯ less than 1x/week	◯ 1-2x/week	◯ more than 2x/week		
**Weight loss**	◯ none	◯ mild	◯ moderate	◯ severe		
**Weight gain**	◯ none	◯ mild	◯ moderate	◯ severe		
**Does your pet ever itch excessively:**	◯ none	◯ lick	◯ chew	◯ head shake	◯ rub	◯ scratch
**How often is your pet itchy:**	◯ none	◯ mild	◯ moderate	◯ severe		
**Parasite history- including giardia**	◯ none	◯ yes (please specify)				
**Ever had pancreatitis**	◯ none	◯ yes (please specify)				
**Any pre- existing conditions**	◯ none	◯ yes (please specify)				
**Any disease as a puppy (ex: Parvovirus, etc.)**	◯ none	◯ yes (please specify)				

Is there anything else that you would like to add or let us know about your pet?
