# Supplementary material for: Developmental stages in microbiota, bile acids, and clostridial species in healthy puppies
Source: J Vet Intern Med. 2020 Oct 13;34(6):2345–56. doi: 10.1111/jvim.15928 (PMC7694855; doi:10.1111/jvim.15928)
Supplement: Supplementary file 2 — Supplemental Table 1 A table listing statistical test results for all variables. Xlsx format. [file JVIM-34-2345-s002.pdf]

	>52 weeks	1-2 weeks	3-4 weeks	5-6 weeks	7-9 weeks	10-16 weeks	20-48 weeks
total bacteria	11.08 [10.65-11.25]	11.13 [7.6-11.45]; p>.999	11.28 [11.09-11.53]; **p=.045**	11.32 [11.04-11.56]; **p=.020**	11.18 [10.74-11.41]; p>.999	10.63 [10.40-11.20]; p=.170	10.90 [10.27-11.2]; p>.999
Faecalibacterium spp.	7.34 [3.81-8.19]	3.80 [2.70-4.59]; **p<.001**	2.93 [2.48-4.19]; **p<.001**	4.28 [1.07-7.69]; **p=.016**	6.40 [1.07-7.63]; p=.506	6.35 [4.62-7.36]; p=.585	7.08 [5.04-7.73]; p>.999
Turicibacter spp.	6.81 [5.31-7.82]	3.22 [2.31-6.33]; **p<.001**	4.12 [2.7-5.17]; **p<.001**	5.41 [4.33-8.35]; p>.999	7.54 [6.14-8.54]; p>.999	5.45 [4.77-8.03]; p=.782	6.64 [5.40-7.94]; p>.999
Streptococcus spp.	3.03 [1.66-5.98]	5.18 [1.97-7.53]; p=.178	5.58 [1.45-8.13]; **p=.027**	8.00 [6.35-8.96]; **p<.001**	6.03 [3.60-7.47]; **p=.007**	4.22 [2.98-7.37]; p>.999	4.13 [1.94-6.58]; p>.999
Escherichia coli	4.19 [.88-8.06]	8.53 [1.65-9.14]; **p=.005**	8.51 [7.90-9.11]; **p<.001**	7.48 [6.15-8.69]; **p=.018**	7.12 [5.42-8.72]; **p=.027**	5.39 [3.46-7.17]; p>.999	5.01 [2.84-6.93]; p>.999
Blautia spp.	10.18 [9.66-10.64]	7.92 [6.96-10.08]; **p<.001**	9.96 [8.42-10.67]; p>.999	10.52 [9.2-11.15]; p>.999	10.30 [9.50-11.04]; p>.999	9.86 [9.49-10.70]; p>.999	10.14 [9.21-10.75]; p>.999
Fusobacterium spp.	9.45 [6.05-10.14]	8.41 [4.92-9.1]; **p=.003**	8.26 [7.58-10.03]; p=.229	8.45 [7.18-10.05]; p=.111	8.94 [6.61-9.90]; p=.220	8.40 [7.73-9.65]; p=.157	8.91 [6.69-9.74]; p=.822
Clostridium hiranonis	6.02 [5.06-7.00]	.01 [.01-5.51]; **p<.001**	.01 [.01-.68]; **p<.001**	.84 [.01-6.71]; **p=.005**	6.36 [.01-6.96]; p>.999	6.23 [.01-6.78]; p>.999	6.29 [5.47-6.96]; p>.999
Dysbiosis Index	-4.62 [-8.36--.61]	5.28 [2.13-8.27]; **p<.001**	6.69 [1.88-8.83]; **p<.001**	5.39 [1.32-8.6]; **p<.001**	-.59 [-3.41-6.01]; p=.052	-3.47 [-6.56-5.01]; p>.999	-4.09 [-6.75-.2]; p>.999
C. perfringens NetF toxin	undetectable in all samples						
C. perfringens 16S rRNA	1.13 [1.13-5.28]	6.48 [2.51-7.75]; **p<.001**	6.26 [4.96-7.63]; **p<.001**	6.53 [2.92-7.86]; **p<.001**	5.69 [2.92-8.33]; **p=.002**	2.54 [1.13-4.62]; p>.999	3.23 [1.13-5.11]; p>.999
C. difficile 16S rRNA	.01 [.01-.01]	3.96 [.01-5.24]; **p<.001**	4.85 [3.86-5.86]; **p<.001**	4.1 [.01-4.85]; **p<.001**	.01 [.01-2.18]; p>.999	.01 [.01-.01]; p>.999	.01 [.01-.01]; p>.999
C. perfringens enterotoxin (CPE)	1.84 [1.84-8.15]	1.84 [1.84-1.84]; **p=.014**	1.84 [1.84-6.21]; p=.305	1.84 [1.84-1.84]; **p=.017**	1.84 [1.84-3.85]; p=.279	1.84 [1.84-3.89]; p=.523	2.29 [1.84-5.30]; p>.999
Salmonella spp.	.01 [.01-.01]	.01 [.01-.01]; p>.999	.01 [.01-.01]; p>.999	.01 [.01-3.21]; p=.612	.01 [.01-3.33]; p=.078	not measured	not measured
Campylobacter jejuni	undetectable in all samples						
Cholic Acid	.13 [.01-1.88]	.59 [.06-4]; p=.500	1.34 [.12-4.2]; **p=.003**	2.12 [.8-5.61]; **p<.001**	.65 [.05-3.08]; p=.173	.22 [.03-1.76]; p>.999	.42 [.03-3.56]; p>.999
Chenodeoxycholic Acid	.07 [.01-.4]	.26 [.01-.78]; p=.670	.37 [.09-2.62]; **p=.021**	1.24 [.54-1.79]; **p<.001**	.70 [.09-1.95]; **p<.001**	.18 [.01-1.00]; p=.811	.36 [.01-1.12]; p>.999
Lithocholic Acid	.71 [.04-1.38]	.04 [.02-.12]; **p<.001**	.05 [.02-.07]; **p<.001**	.06 [.04-.97]; **p=.050**	.75 [.02-1.36]; p>.999	.36 [.05-1.70]; p>.999	.38 [.16-.92]; p>.999
Deoxycholic Acid	3.53 [.24-7.54]	.22 [.07-.28]; **p<.001**	.24 [.08-.30]; **p<.001**	.26 [.22-3.75]; **p=.001**	2.42 [.22-6.08]; p>.999	1.68 [.22-4.09]; p=.912	1.91 [1.00-3.51]; p>.999
Ursodeoxycholic Acid	.02 [0-.08]	.03 [0-.44]; p>.999	.19 [.01-1.51]; **p=.003**	.26 [.01-.82]; **p<.001**	.23 [.02-.91]; **p<.001**	.06 [0-.30]; p>.999	.08 [.01-.16]; p>.999

Total Primary Bile Acid	.26 [.03-2.14]	.90 [.08-4.43]; p=.481	1.78 [.20-6.82]; **p=.003**	3.27 [1.34-7.23]; **p<.001**	1.32 [.14-5.03]; **p=.017**	.40 [.05-2.56]; p>.999	.78 [.04-4.67]; p>.999
Total Secondary Bile Acid	4.12 [.32-8.94]	.29 [.10-.75]; **p<.001**	.45 [.13-1.86]; **p<.001**	.61 [.28-5.06]; **p=.006**	3.48 [.82-7.94]; p>.999	1.94 [.56-5.85]; p>.999	2.30 [1.47-4.33]; p>.999
Total Bile Acid	5.09 [2.04-9.31]	1.34 [.18-4.58]; **p<.001**	2.34 [.78-7.29]; p=.064	5.06 [1.87-8.31]; p>.999	4.92 [3.77-9.09]; p>.999	3.12 [1.82-6.00]; p=.637	5.10 [1.86-6.26]; p>.999
% Secondary Bile Acid	95.43 [13.02-99.22]	22.13 [3.29-82.61]; **p<.001**	20.57 [6.44-74.01]; **p<.001**	15.44 [5.85-60.89]; **p<.001**	75.80 [16.58-96.46]; p=.375	91.57 [18.05-97.52]; p>.999	79.05 [25.36-98.16]; p>.999
% Primary Bile Acid	4.57 [.79-86.98]	77.87 [17.4-96.71]; **p<.001**	79.43 [25.99-93.56]; **p<.001**	84.56 [39.11-94.15]; **p<.001**	24.20 [3.54-83.42]; p=.375	8.43 [2.48-81.95]; p>.999	20.95 [1.84-74.64]; p>.999
% Cholic Acid	2.31 [.44-76.52]	43.22 [15.25-87.4]; **p=.001**	60.32 [15.1-79.32]; **p<.001**	54.61 [20.47-74.24]; **p<.001**	11.77 [1.17-51.91]; p>.999	5.67 [.64-53.91]; p>.999	10.42 [1.26-56.78]; p>.999
% Chenodeoxycholic Acid	1.89 [.2-10.46]	15.19 [1.84-59.93]; **p<.001**	20.8 [7.61-35.96]; **p<.001**	28.74 [18.64-35.98]; **p<.001**	13.61 [2.38-32.42]; **p=.003**	3.77 [.58-29.36]; p=.343	7.04 [0.58-17.86]; p>.999
% Lithocholic Acid	13.8 [1.7-34.87]	3.22 [.39-14.66]; **p=.036**	1.59 [.63-3.38]; **p<.001**	1.43 [.71-11.67]; **p<.001**	13.7 [.32-27.98]; p>.999	14.17 [1.54-33.77]; p>.999	15.17 [2.49-27.99]; p>.999
% Deoxycholic Acid	74.45 [9.77-85.73]	13.01 [2.49-37.62]; **p<.001**	6.61 [2.74-13.71]; **p<.001**	6.71 [3.44-45.11]; **p<.001**	49.61 [4.55-67.22]; p=.133	59.43 [7.15-85.05]; p>.999	62.51 [21.53-81.81]; p>.999
% Ursodeoxycholic Acid	.36 [.03-3.16]	2.48 [.36-40.26]; **p=.014**	6.95 [.12-59.83]; **p<.001**	4.11 [.55-16.18]; **p=.001**	4.69 [.30-18.69]; **p<.001**	.98 [.07-9.36]; p>.999	1.24 [.29-8.41]; p>.999

Bile acid concentrations (median [min-max] μg/mg lyophilized feces), bile acid proportions (median [min-max] % of total bile acids), abundance of bacterial groups and toxins (median [min-max] LogSQ), and Dysbiosis Index (median [min-max]) in young dog age groups compared to adult dogs (>52 weeks) with Dunn's post test for multiple comparisons.
