# Supplementary material for: Developmental stages in microbiota, bile acids, and clostridial species in healthy puppies
Source: J Vet Intern Med. 2020 Oct 13;34(6):2345–56. doi: 10.1111/jvim.15928 (PMC7694855; doi:10.1111/jvim.15928)
Supplement: Supplementary file 3 — Supplemental Figure 1 Abundance (log DNA) of total bacteria. Red lines indicate medians and red asterisks indicate significance in comparison to adult dogs (ie, dogs >52 weeks of age; P < .05). [file JVIM-34-2345-s003.pdf]

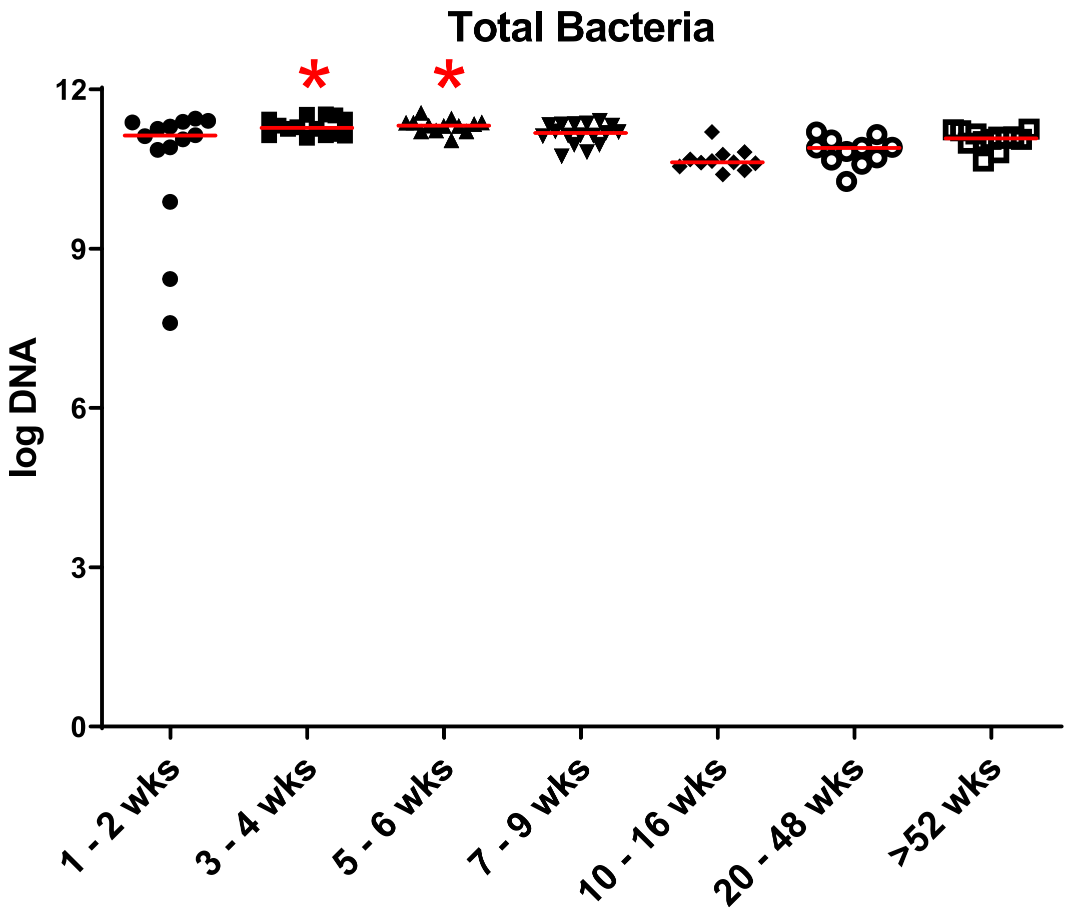

Supplemental Figure 1. Abundance (log DNA) of total bacteria. Red lines indicate medians and red asterisks indicate significance in comparison to adult dogs (i.e., dogs > 52 weeks of age; $p < .05$).
