# Supplementary material for: Developmental stages in microbiota, bile acids, and clostridial species in healthy puppies
Source: J Vet Intern Med. 2020 Oct 13;34(6):2345–56. doi: 10.1111/jvim.15928 (PMC7694855; doi:10.1111/jvim.15928)
Supplement: Supplementary file 5 — Supplemental Figure 3 Concentration of fecal bile acids (μg/mg lyophilized feces). Red lines indicate medians and red asterisks indicate significance in comparison to adult dogs (ie, dogs >52 weeks of age; P < .05). [file JVIM-34-2345-s005.pdf]

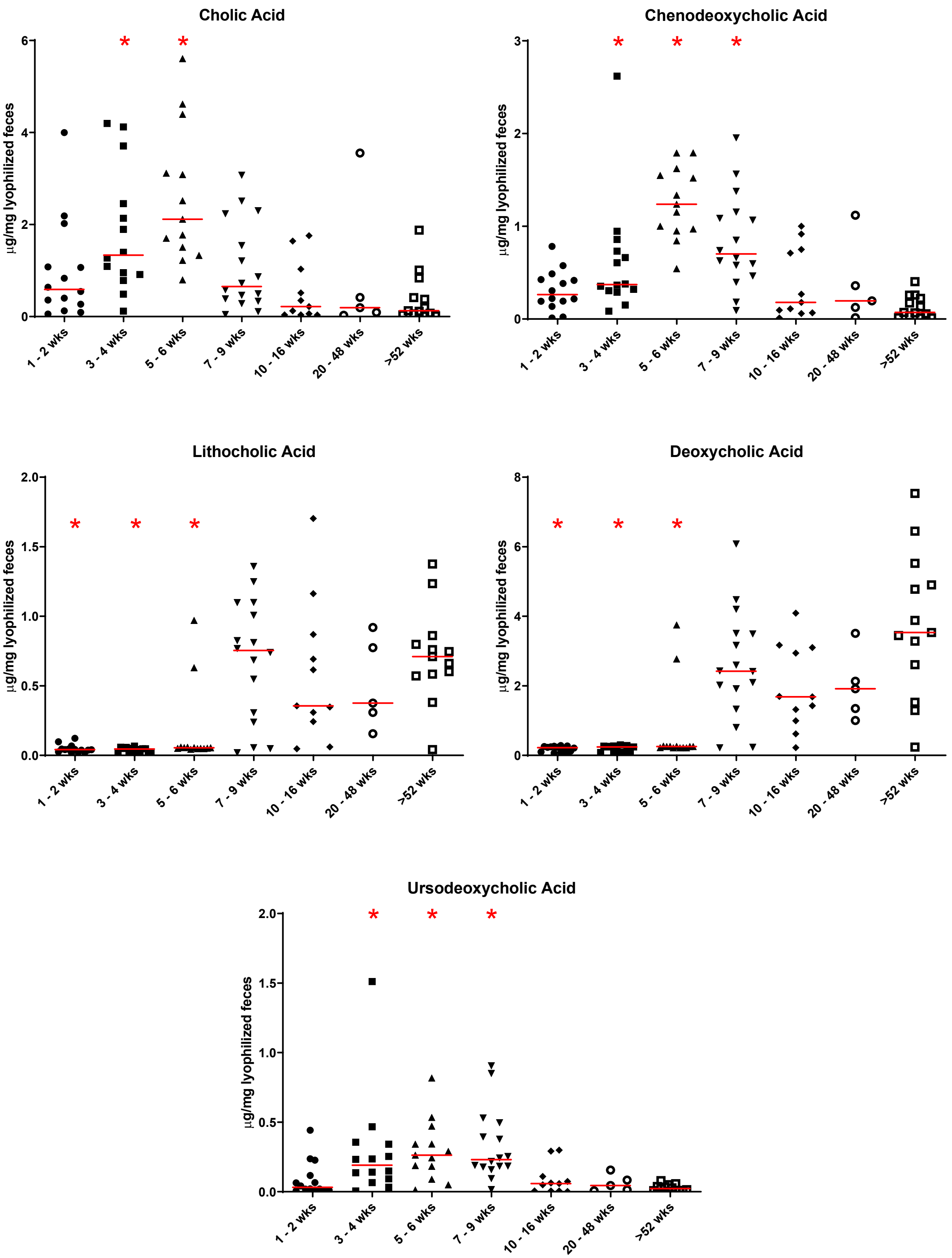

Cholic Acid
Chenodeoxycholic Acid
Lithocholic Acid
Deoxycholic Acid
Ursodeoxycholic Acid
μg/mg lyophilized feces
1 - 2 wks
3 - 4 wks
5 - 6 wks
7 - 9 wks
10 - 16 wks
20 - 48 wks
>52 wks
*

Supplemental Figure 3. Concentration of fecal bile acids (µg/mg lyophilized feces). Red lines indicate medians and red asterisks indicate significance in comparison to adult dogs (i.e., dogs > 52 weeks of age; $p < .05$).
